# Clinical Outcomes and Success Factors of Pterygoid Implants in the Posterior Atrophic Maxilla: A Prospective Study

**DOI:** 10.7759/cureus.82820

**Published:** 2025-04-23

**Authors:** Waseem F Mirdah, Rohit Goyal, Aakanksha Singh, Navneet Singh, Shakya K Laxmi, Kenil J Tarpara, Debasmita Acharjee

**Affiliations:** 1 Department of Oral and Maxillofacial Surgery, Maharaja Ganga Singh Dental College and Research Centre, Sri Ganganagar, IND; 2 Department of Oral and Maxillofacial Surgery, Surendera Dental College and Research Institute, Sri Ganganagar, IND

**Keywords:** atrophic, bone loss, implants, maxilla, pterygoid, success

## Abstract

Introduction

Pterygoid implants serve as an alternative for rehabilitating a posterior atrophic maxilla without requiring extensive bone grafting or sinus augmentation. This study aimed to evaluate the clinical outcomes and success rates of pterygoid implants over a one-year follow-up period and assess the influence of bone quality, complications, and other patient-related factors on implant success.

Materials and methods

A total of 34 patients with a posterior atrophic maxilla received 35 pterygoid implants. Preoperative cone-beam computed tomography (CBCT) was used to assess bone quality, and the implants were placed using a standardized surgical protocol. Clinical parameters, including primary stability, marginal bone loss, postoperative complications, and patient-reported outcomes, were recorded. Implant success was defined as the absence of pain, mobility, radiographic bone loss beyond 1.5 mm during one-year follow-up, or infection. Patient-reported outcomes were evaluated using the Oral Health Impact Profile (OHIP-14). Statistical analysis was conducted to determine the correlation between implant success and influencing factors such as bone density, complications, smoking history, age, and implant length.

Results

The overall success rate was 31 (88.57%) pterygoid implants within the one-year follow-up period. Bone quality significantly affected implant success, with D3 showing a higher failure rate than D2 (p = 0.029). Complications, including implant fracture, prosthetic failure, and nerve injury, were significantly associated with implant failure (p = 0.001). Marginal bone loss was higher in the failed implants, supporting its role as a predictive factor of long-term success. Patient age, sex, smoking history, and implant length did not significantly influence outcomes. OHIP-14 scores indicated that patients with successful implants reported improved function and quality of life.

Conclusion

The pterygoid implants demonstrated a high success rate and served as a viable treatment for posterior maxillary rehabilitation. Bone quality and complications were the key determinants of implant success, whereas age, sex, smoking history, and implant length had no significant impact. Marginal bone loss has emerged as a crucial factor for implant failure, highlighting the importance of postoperative monitoring.

## Introduction

Rehabilitation of an atrophic posterior maxilla poses a significant challenge within the realm of implant dentistry, primarily attributed to the reduction in both the height and volume of the alveolar bone, often exacerbated by sinus pneumatization [1]. In such circumstances, conventional implant placement can be considerably affected, necessitating the exploration of alternative strategies that ensure long-term functionality and stability [2]. One such strategy is the utilization of pterygoid implants, which are affixed to the pterygoid region of the maxilla, consequently providing prosthetic restorations with enhanced support [3]. Pterygoid implants have been acknowledged as an advantageous solution for individuals with pronounced maxillary atrophy, thereby eliminating the need for complex bone grafting procedures while simultaneously yielding improved prosthetic results [4].

The primary rationale behind the use of pterygoid implants is their ability to bypass the maxillary sinus and engage the dense cortical bone of the pterygoid region. This anatomical advantage not only facilitates successful implant osseointegration but also reduces the overall treatment duration compared with sinus augmentation procedures [3,4]. Furthermore, pterygoid implants contribute to a more even distribution of occlusal forces, thereby improving prosthetic longevity and patient comfort. As implantology advances, evaluating the success rates and clinical outcomes of these implants becomes crucial in determining their efficacy as a standard treatment modality for the rehabilitation of the atrophic posterior maxilla. Araujo et al. reported a high success rate and concluded that pterygoid implants could be successfully used in atrophic posterior maxilla [5].

Although pterygoid implants have been documented in the literature for their clinical success [5], comprehensive studies examining their long-term outcomes and patient-centered benefits are still limited. The novelty of this study lies in its prospective design, detailed evaluation of clinical and demographic factors, and emphasis on patient-reported outcomes. This study aimed to evaluate the success rates of pterygoid implants and prostheses in correlation with various clinical and demographic variables. This study focused on assessing the efficacy and safety of pterygoid implants for the rehabilitation of the atrophic posterior maxilla, providing clinicians with valuable insights to aid in treatment decisions. Additionally, it evaluated the cumulative success rates of pterygoid implants and their prostheses, considering their long-term viability. Furthermore, potential complications associated with the procedure, such as infection, nerve damage, and implant fracture, were analyzed to identify the risk factors and develop strategies for their prevention.

## Materials and methods

Study design and setting

This prospective, quasi-experimental study was conducted on patients who visited the Department of Oral and Maxillofacial Surgery, Maharaja Ganga Singh Dental College, Sri Ganganagar, Rajasthan, and required rehabilitation of the posterior atrophic maxilla with pterygoid implants between June 2023 and January 2025. The study was approved by the Institutional Ethics Committee (MGSDC/SY/23/6) and adhered to the principles of the Declaration of Helsinki. Written informed consent was obtained from all patients.

Patient’s eligibility

The study included patients with good general health and no contraindications for surgery, a minimum bone width of 6 mm at the implant site, no sinusitis symptoms, and no history of sinus floor augmentation or bone graft procedures. A minimum follow-up period of one year was maintained after implant placement. Patients with systemic medical conditions (American Society of Anesthesiologists (ASA) II or higher), a history of bisphosphonate therapy, acute infections, or failed primary implant stability (less than 70 N/cm) were excluded.

Sample size estimation

Sample size estimation was performed using G*Power software version 3.6.9 (Heinrich-Heine-Universität Düsseldorf, Düsseldorf, Germany). Using an a priori calculation for a one-sample proportion test with 95% power and a 5% alpha error, the estimated minimum sample size was 29, based on an expected success rate of 95% for pterygoid implants [6]. Considering a 20% loss to follow-up, the sample size was increased to 34 patients.

Methodology

**Figure 1:**
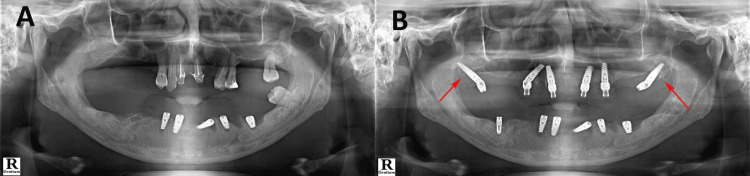
Orthopantomographic (OPG) image (A) Pterygoid region before implant placement, (B) After pterygoid implant placement This OPG image is of a patient from the study and is used with the permission of the patient.

A total of 34 patients (20 males, 14 females) were treated by the same oral surgeon with more than 10 years of experience using the same standardized technique. All the patients received unilateral pterygoid implants, except one who received bilateral pterygoid implants (Figure 1).

**Figure 2:**
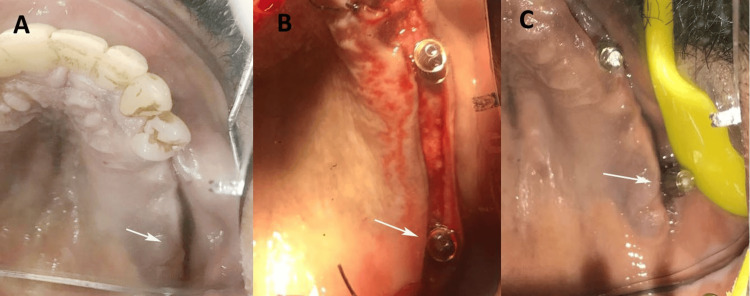
(A) Preoperative edentulous left maxillary posterior region, (B) Surgical placement of pterygoid implant, (C) Postoperative follow-up after one month This figure is of a patient from the study and is used with the permission of the patient.

Therefore, 35 pterygoid implants were placed in 34 patients. The surgical procedure involved local anesthesia using 2% lignocaine with 1:100,000 adrenaline. The Nobel Biocare Pterygoid Implant System (Nobel Biocare, Switzerland) was used, with implant diameters of 4.2 mm and lengths of 15 and 18 mm, placed at an angulation to engage the pterygoid region. Additionally, the Straumann Bone Level Tapered (BLT) Implant System with a Laser-Lok surface (Straumann Int., Switzerland) was employed to enhance osseointegration and minimize the risk of peri-implantitis. To insert the pterygoid implants, the maxillary tuberosity was accessed through a crestal incision. The implant site was prepared using a straight handpiece for drilling in a 45^0^ posterior and superior orientation, and 45^0^ to the palate to accurately determine the trajectory of the implant axis. In instances where the implants were placed at a reduced angle, a contra-angled handpiece was employed to maintain a predominantly vertical alignment. The drilling sequence was carefully executed to ensure the accurate positioning of the implant. Initially, a 2.0 mm pilot drill was used at 800-1200 rpm with copious irrigation to create the initial osteotomy trajectory. This was followed by sequential widening with the 2.8 mm and 3.5 mm twist drills at a reduced speed of 600-900 rpm to prevent overheating of the bone. The angulation was confirmed by an intraoperative radiograph. Final osteotomy preparation with a 4.2 mm cortical drill was performed at 400-600 rpm, ensuring adequate engagement of the cortical bone while maintaining bone integrity. To enhance the primary stability, bicortical engagement of the pterygoid plate was achieved by carefully controlling the drilling depth and angulation. The pterygoid implant was then placed at a slow speed using a torque-controlled handpiece at the recommended torque of 35-45 N/cm. The final implant positioning was verified radiographically before proceeding with the placement of the cover screw (Figure 2).

After implant placement, a cover screw was positioned, and primary closure was achieved using 4-0 Vicryl sutures (Ethicon, Johnson & Johnson, Raritan, NJ, US). All the patients received postoperative prescriptions, including a combination of 500 mg of amoxicillin and 125 mg of potassium clavulanate thrice daily for seven days, a non-steroidal anti-inflammatory drug (ibuprofen 600 mg) as needed for pain management, and 0.12% chlorhexidine mouthwash twice daily for two weeks. The sutures were removed after 8-10 days, and prosthetic loading was performed 3-4 months post-implant placement. After the healing period, straight or angulated abutments were placed according to the case requirement, followed by the placement of the fixed prostheses.

Outcome assessment

Measurements and evaluations included bone quality assessment using the Kodak Carestream cone-beam computed tomography (CBCT) scanner (Carestream Health, Rochester, NY, US). Bone quality was categorized as D2 and D3 [7]. D2 bone has a thick layer of dense cortical bone with dense trabecular bone inside, offering strong support, typically seen in the anterior maxilla and posterior mandible. In contrast, D3 bone has a thinner cortical layer and less dense trabecular bone, making it softer and more common in the posterior maxilla. Marginal bone loss was assessed as the straight-line distance from the implant apex to the crestal bone on both the mesial and distal aspects. The patient’s perception was checked using the Oral Health Impact Profile (OHIP)-14 questionnaire [8], in which 14 questions were scored using a four-point Likert scale. The complication rates were recorded based on nerve damage, implant fractures, and prosthetic failures.

Clinical success parameters were determined based on implant survival rate, marginal bone loss, patient satisfaction in terms of masticatory function and speech improvement, and absence of significant complications such as peri-implantitis, fractures, or infections. This methodology ensures a structured and comprehensive evaluation of pterygoid implants, integrating advanced diagnostic tools, standardized surgical protocols, and postoperative monitoring systems to optimize patient outcomes. All patients were followed up for a period of one year after prosthetic placement. A successful implant is defined by the absence of clinical mobility, pain, discomfort, or infection, along with the lack of peri-implant radiolucency on radiographic evaluation. Marginal bone loss should not exceed 1.5 mm during the first year. The implant must support a stable, functional prosthesis without mechanical complications such as loosening or fracture [9].

To ensure the accuracy and reliability of the measurements, the clinician performing the evaluations carried out calibration sessions using standard measurement protocols. Inter- and intra-examiner reliability were assessed through repeated measurements of test cases, ensuring consistency in bone density readings, implant stability scores, and radiographic assessments. The devices used were calibrated before each evaluation session according to the manufacturer’s guidelines to maintain measurement precision and validity.

Statistical analysis

Data analysis was performed using the Stata Statistical Software Release 18 (StataCorp LLC, College Station, TX, USA). The normality of the data was assessed and verified using the Kolmogorov-Smirnov test. Categorical variables are expressed as frequencies and percentages, while continuous variables are reported as means and standard deviations. Bivariate analyses were conducted to evaluate the relationships between continuous and categorical variables as well as between pairs of categorical variables. Pearson correlation coefficients were calculated to examine the associations among continuous variables. Statistical significance was established at a p-value < 0.05.

## Results

The sample comprised 20 males (58.82%) and 14 females (41.18%). The mean age for males was 56.25 ± 6.24 years, while the mean age for females was 47.4 ± 7.62 years. The overall age distribution indicated a slightly older male cohort than the female cohort (Table 1).

**Table 1:** Distribution of sex and mean age of study participants The number of males and females is represented in the form of N (%), and age is represented in the form of mean ± standard deviation (SD).

Sex	N (%)	Mean ± SD	Minimum	Maximum
Male	20 (58.82)	56.25 ± 6.24	45	65
Female	14 (41.18)	47.40 ± 7.62	34	62

Out of 35 (100%) pterygoid implants placed in 34 patients, 4 (11.43%) implants failed during the one-year follow-up, and 31 (88.57%) implants were successful. Sex distribution showed no significant difference (p = 0.066), with males and females having comparable outcomes. Bone quality was significant (p = 0.029), with D3 being linked to more failures than D2. Complications were highly significant (p = 0.001), as all successes had no complications, whereas failures included nerve damage, implant fracture, and prostheses failure. Smoking history had no significant effect (p = 0.664). These findings suggest that bone quality and complications, unlike sex or smoking, strongly influence success (Table 2).

**Table 2:** Bivariate analysis for the association between categorical variables using the chi-square test of association *p-value < 0.05: significant Data are presented in the form of N (%).

Variables	Outcomes	Success N (%)	Failure N (%)	Chi-test	p-value
		31 (88.57%)	4 (11.43%)		
Sex	Male	16 (45.71%)	4 (11.43%)	3.39	0.066
	Female	15 (42.86%)	0 (0%)		
Bone quality	D2	18 (51.43%)	0 (0%)	4.78	0.029*
	D3	13 (37.14%)	4 (11.43%)		
Complications	Nil	31 (88.57%)	0 (0%)	35.00	0.001*
	Nerve damage	0 (0%)	2 (5.71%)		
	Failure of prostheses	0 (0%)	1 (2.86%)		
	Fracture of implants	0 (0%)	1 (2.86%)		
Past history of smoking	Yes	19 (54.29%)	2 (5.71%)	0.19	0.664
	No	12 (34.29%)	2 (5.71%)		

Bivariate analysis comparing the means of continuous variables between successful and failed pterygoid implants revealed significant differences in marginal bone loss but not in age or implant length. Age showed no significant association with success, suggesting that it did not influence the outcomes. Similarly, implant length was comparable between the groups, indicating no impact on success rates. Patient perception, as assessed from their OHIP scores, revealed statistically significantly higher scores in patients with successful implants than those with failed implants. Similarly, marginal bone loss was significantly higher in the failure group, which indicated that greater marginal bone loss, particularly mesial and average, and perception of the patients strongly correlated with implant failure, whereas age and implant length did not appear to be determining factors in this cohort of 34 patients (Table 3).

**Table 3:** Bivariate analysis for the comparison of the mean for continuous variables using the independent t-test *p-value < 0.05: significant Data are presented in the form of mean ± standard deviation (SD). OHIP: Oral Health Impact Profile

Variables	Success or failure of implants	95% CI for mean	Mean ± SD	t-stat	p-value
Age (years)	Success	49.17 - 55.15	52.16 ± 8.15	0.61	0.551
	Fail	41.54 - 67.96	54.75 ± 8.30		
Implant length (mm)	Success	18.69 - 20.02	19.35 ± 1.82	0.85	0.401
	Fail	14.50 - 22.50	18.50 ± 2.52		
Marginal bone loss on mesial (mm)	Success	0.51 - 0.72	0.62 ± 0.29	4.20	0.001*
	Fail	0.73 - 1.83	1.28 ± 0.35		
Marginal bone loss on distal (mm)	Success	0.66 - 0.87	0.76 ± 0.29	2.74	0.01*
	Fail	0.80 - 1.56	1.18 ± 0.24		
Average bone loss (mm)	Success	0.60 - 0.78	0.69 ± 0.25	4.16	0.001*
	Fail	1.00 - 1.46	1.23 ± 0.15		
OHIP-14 score	Success	22.25 – 43.55	32.90 ± 10.65	25.43	0.001*
	Fail	15.43 – 33.37	24.40 ± 8.97		

## Discussion

The present study aimed to assess the clinical outcomes and success rates of pterygoid implants in patients with a posterior atrophic maxilla over a one-year follow-up period. The findings suggested an overall success rate of 31 (88.57%) pterygoid implants. Araujo et al. evaluated the clinical outcomes of pterygoid implants and reported a high success rate of 1795 (94.87%) pterygoid implants in six studies [5]. Similar findings were reported in a systematic review by D'Amario et al. during 1-6 years of follow-up [10]. This supports the reliability of pterygoid implants as a viable alternative for rehabilitating posteriorly atrophic maxilla, particularly in cases where sinus floor augmentation or extensive bone grafting may not be preferred. The absence of significant differences in success rates between males and females suggests that sex is not a determining factor.

Influence of bone quality

Bone quality emerged as a significant determinant of implant success, with the D3 bone exhibiting a higher failure rate than the D2 bone (p = 0.029). This finding corroborates previous research indicating that denser bone provides better primary stability and osseointegration [11]. The pterygoid region, characterized by dense cortical bone engagement, is generally considered favorable for implant placement [3,4]. However, in cases where the D3 bone predominates, primary stability may be compromised, increasing the risk of micromovements and implant failure. Therefore, preoperative bone quality assessment using CBCT should be emphasized to aid case selection and surgical planning.

Complications and their impact on the success rate

The presence of complications was highly significant (p = 0.001) in determining implant failure. Among the failed implants, documented complications included nerve damage, implant fracture, and prosthetic failure. The importance of meticulous surgical planning and execution cannot be overstated, as minor deviations in angulation or depth can lead to adverse outcomes. Implant fractures, although rare, highlight the importance of ensuring optimal torque application and stress distribution [5]. However, prosthetic failures may stem from biomechanical complications or improper occlusal adjustments, reinforcing the need for precise prosthetic planning [6].

Marginal bone loss and implant failure

A key finding of this study was the significant association between marginal bone loss and implant failure. An average marginal bone loss of 0.6-1.46 mm was reported in the present study. Curi et al. reported a mean marginal bone loss of 0.31-1.75 mm over a follow-up period of 3 years [6]. Peñarrocha et al. reported a mean marginal bone of 0.71 mm during one year of follow-up [12]. Failed implants demonstrated greater marginal bone loss than successful implants, indicating that early bone loss may be a precursor of implant failure. This aligns with the literature, where excessive marginal bone loss has been linked to biomechanical overload, inflammatory responses, and peri-implantitis [13,14]. Regular radiographic monitoring and patient adherence to postoperative maintenance protocols are essential for minimizing marginal bone loss and enhancing implant longevity.

Patient perception and quality of life

OHIP-14 scores demonstrated that patients with successful implants reported significantly higher satisfaction levels in terms of masticatory function and speech improvement. This underscores the impact of pterygoid implants not only on functional rehabilitation but also on patient-reported outcomes. The psychological and social aspects of dental rehabilitation play a crucial role in the overall treatment success, reinforcing the importance of patient-centered approaches in implant dentistry [15]. Similar findings have been reported by Peñarrocha et al. [12].

Effect of smoking on implant success

Contrary to some studies reporting the negative effects of smoking on implant success due to impaired healing and increased peri-implant bone loss [16-18], the present study found no significant association between smoking history and implant success (p = 0.664). This finding could be attributed to the small sample size and the fact that all patients were former smokers who had ceased smoking prior to the implantation phase. However, clinicians should remain cautious, as larger studies have consistently shown higher failure rates in smokers than in non-smokers.

Influence of age and implant length

Age and implant length did not significantly affect implant success. The lack of age-related differences is in agreement with other studies suggesting that chronological age alone is not a contraindication for implant therapy, as long as systemic health permits [19]. According to a study by Bertl et al., patients older than 65 years are at a higher risk of implant failure [19]. In our study, all patients were younger than 65 years of age, with a mean age of 56 years. This could have been the reason why age was not associated with implant failure in the present study. Similarly, implant length was not a determining factor, which is consistent with findings that primary stability and osseointegration are influenced more by implant design and surgical technique than by implant length alone [5]. The angulation and engagement of the cortical bone in the pterygoid region appear to compensate for any potential disadvantages associated with implant length.

Clinical implications

The findings of this study reinforce the efficacy of pterygoid implants as a predictive treatment modality for posterior maxillary rehabilitation. Preoperative assessment of bone quality using CBCT is an essential part of treatment planning to identify cases at a higher risk of failure. Additionally, maintaining strict surgical protocols, minimizing biomechanical stress, and ensuring patient compliance with postoperative care are crucial for optimizing outcomes. The study also highlights the importance of considering patient-reported outcomes, as functional and psychological satisfaction significantly influence treatment success.

Limitations and future recommendations

Despite its strengths, this study had several limitations. The relatively small sample size limits the generalizability of the findings, and larger multicenter trials with longer follow-up durations are required to confirm these results. Additionally, while bone quality was assessed using CBCT, future studies should explore more detailed microstructural bone analyses, such as finite element modeling, to better understand the biomechanical properties that influence implant success. Another limitation is the one-year follow-up period. While short-term success has been demonstrated, long-term data on implant survival, prosthetic complications, and peri-implant health are necessary to draw more definitive conclusions. Further research should investigate the effects of prosthetic design variations and loading protocols on pterygoid implant longevity.

## Conclusions

This study demonstrated a success rate of 31 (88.57%) pterygoid implants in rehabilitating the posterior atrophic maxilla. Bone quality significantly influenced outcomes, with D3 bone linked to higher failure rates, whereas patient age, sex, smoking status, and implant length had no significant impact. Marginal bone loss is a key predictor of failure, which reinforces the importance of postoperative monitoring. Complications such as nerve damage, implant fractures, and prosthetic failures are significantly associated with implant loss. Patients with successful implants reported significantly better functional and psychological satisfaction, emphasizing the role of pterygoid implants in improving the quality of life. Future research should focus on larger cohorts and long-term follow-up to refine clinical protocols and enhance success rates. Strict adherence to surgical protocols and postoperative care is crucial for optimal outcomes.
